# Innominate artery versus combined innominate and femoral artery perfusion in acute type A aortic dissection: a comparison of surgical efficiency and early outcomes

**DOI:** 10.3389/fcvm.2026.1808621

**Published:** 2026-07-06

**Authors:** Lina Wu, Li Liu, Jun Wei, Yang Zhang, Fangyuan Chen, Yun Lu

**Affiliations:** 1 Department of Cardiothoracic Surgery, Affiliated Hospital of Xuzhou Medical University, Xuzhou, China; 2 Department of Cardiac Surgery, XuZhou Clinical School of Xuzhou Medical University, Xuzhou, China; 3 Department of Cardiology, The Second Affiliated Hospital of Xuzhou Medical University, Xuzhou, China

**Keywords:** acute type A aortic dissection, cardiopulmonary bypass, femoral artery perfusion, innominate artery antegrade perfusion, postoperative complications, surgical efficiency

## Abstract

**Objective:**

This study aimed to compare the impact of isolated innominate artery antegrade perfusion (IA) versus combined innominate and femoral artery perfusion (IA + FA) on surgical efficiency and short-term outcomes in patients undergoing surgery for acute type A aortic dissection (ATAAD).

**Methods:**

A single-center, retrospective cohort study was conducted involving 94 consecutive ATAAD patients who underwent surgery between May 2024 and May 2025. Patients were allocated into two groups based on the perfusion strategy: the IA group (*n* = 56) and the IA + FA group (*n* = 38). The primary endpoint was 30-day all-cause mortality. Secondary endpoints included total operative duration, aortic cross-clamp time, and the incidence of major postoperative complications (e.g., cerebral infarction, acute kidney injury, re-sternotomy). Statistical analyses were performed to compare intergroup differences.

**Results:**

The two groups were well-balanced in terms of baseline demographics and preoperative risk profiles. The total operative time was significantly shorter in the IA group compared to the IA + FA group (370.05 ± 63.34 min vs. 397.26 ± 62.11 min, *p* = 0.04), while no significant differences were observed in aortic cross-clamp time or deep hypothermic circulatory arrest duration. Regarding safety outcomes, there were no statistically significant differences in 30-day mortality (10.71% vs. 21.05%, *p* = 0.28), postoperative cerebral infarction (14.29% vs. 15.79%, *p* = 1.00), or acute kidney injury (21.43% vs. 26.32%, *p* = 0.76). However, numerical trends favoring the IA group were observed for rates of re-sternotomy and new requirement for hemodialysis.

**Conclusion:**

For ATAAD surgery, an isolated innominate artery antegrade perfusion strategy significantly improves surgical efficiency by reducing operative time without compromising early safety outcomes, compared to a combined IA + FA approach. The routine addition of femoral artery perfusion did not confer measurable clinical benefits in this cohort. Thus, IA should be considered as a streamlined and efficient first-line perfusion strategy, potentially reserving IA + FA for select cases with specific indications. These findings warrant further validation in larger, prospective studies.

## 1 Introduction

Acute type A aortic dissection (ATAAD) is one of the most critical emergencies in cardiovascular surgery, with a mortality rate as high as 50% within 24 h (1). Surgical intervention is the only curative approach, but the management of cardiopulmonary bypass (CPB) during the operation, particularly the selection of perfusion strategies, is directly related to the protection of vital organs such as the brain and kidneys, as well as the patient's survival rate (2).The limitations of traditional femoral artery perfusion (FA): Retrograde blood flow is prone to cause aortic dissection with false lumen expansion, vascular endothelial injury, and increased risks of cerebral embolism and lower limb ischemia (3). The innovation of antegrade perfusion: Innominate Artery Antegrade Perfusion (IA), due to its alignment with the physiological direction of blood flow (True-lumen perfusion), effectively reduces the risk of cerebral embolism and has become a recommended strategy in recent guidelines (1). The combined perfusion of the femoral artery and innominate artery (IA + FA) aims to achieve both cerebral protection and lower limb perfusion. However, the complexity of the procedure may prolong the operation time, and the risk of systemic embolism remains controversial (4).While both IA and IA + FA strategies coexist in clinical practice, high-quality evidence addressing two critical questions remains lacking: 1. Can IA enhance surgical efficiency (e.g., reducing operative/cross-clamp times) while ensuring organ safety? 2. Does the technical complexity of IA + FA counteract its potential benefits? This study aims to provide comparative analysis elucidating the impact of Innominate Artery Antegrade Perfusion (IA) versus Combined Perfusion (IA + FA) on surgical efficiency and short-term outcomes, thereby establishing an evidence-based foundation for individualized perfusion strategies in ATAAD.

## 2 Method

### 2.1 Research design and ethics

#### 2.1.1 Study population

Clinical data from 94 consecutive cases were collected from the Cardiac Surgery Department of Xuzhou Medical University, spanning from May 2024 to May 2025.

Ethical Approval Number: XYFY2025-KL154-01.

This retrospective study was approved by the Institutional Ethics Committee, with a waiver of informed consent granted due to its observational design and use of Retrospective data.

### 2.2 Study participants and group allocation

#### 2.2.1 Inclusion criteria

Participants meeting all of the following conditions were enrolled:

Confirmed diagnosis of acute Type A aortic dissection (DeBakey type I or II); Underwent total arch replacement or hemiarch reconstruction;

Cardiopulmonary bypass (CPB) utilizing moderate hypothermic circulatory arrest (25–28 °C) with concomitant cerebral perfusion (5).

All patients underwent either isolated ascending aortic replacement or total arch replacement with frozen elephant trunk (TAR + FET) implantation.

#### 2.2.2 Exclusion criteria

Individuals were excluded if they had any of the following:

Traumatic or iatrogenic aortic dissection;

Preoperative cardiac arrest requiring CPR;

Non-IA/IA + FA perfusion strategy (e.g, isolated femoral artery perfusion).

Group Allocation Criteria

IA Group: Solely received antegrade perfusion via innominate artery cannulation (*n* = 56).

IA + FA Group: Received combined perfusion with simultaneous innominate artery and femoral artery cannulation (*n* = 38).

#### 2.2.3 Perfusion strategy selection criteria

The decision to employ either the IA or IA + FA perfusion strategy was made intraoperatively by the attending surgical team based on a comprehensive assessment of the patient's anatomical and clinical status. The selection criteria were as follows:

Innominate Artery Antegrade Perfusion (IA) Strategy: This was the preferred and first-line approach. It was selected when the following conditions were met: (1) the innominate artery was confirmed to be free of significant dissection involvement or severe atherosclerosis/calcification upon preoperative imaging and intraoperative inspection; (2) there was no clinical or radiological evidence of severe lower limb malperfusion (e.g., absence of dorsalis pedis pulse, ischemic signs) preoperatively.

Combined Innominate and Femoral Artery Perfusion (IA + FA) Strategy: This strategy was considered and utilized in the following scenarios: (1) when preoperative imaging suggested potential compromise or unfavorable anatomy of the innominate artery for safe cannulation, prompting the need for a backup arterial inflow; (2) in the presence of documented severe lower limb malperfusion preoperatively, where adjunctive femoral artery perfusion was intended to address distal ischemia concurrently; (3) based on the surgical team's preference during certain periods of the study, reflecting evolving institutional protocols. The final allocation of patients into the IA or IA + FA group was strictly based on the perfusion strategy actually implemented during surgery.

### 2.3 Variable definitions and data sources

#### 2.3.1 Primary endpoint

Thirty-day mortality was defined as all-cause death occurring within 30 days postoperatively. Verification was performed through linkage between institutional electronic medical records and the national vital statistics registry.

#### 2.3.2 Secondary endpoints

#### 2.3.3 Surgical efficiency metrics

Total operative duration: Time interval (minutes) measured from anesthesia induction to complete skin closure.

Aortic cross-clamp time: Cumulative duration (minutes) between application and removal of the aortic cross-clamp.

Total cardiopulmonary bypass time.

#### 2.3.4 Organ dysfunction

#### 2.3.5 Acute kidney injury (AKI)

Diagnosed according to KDIGO criteria as an absolute increase in serum creatinine ≥0.3 mg/dL within 48 h postoperatively (6).

#### 2.3.6 Postoperative cerebral infarction

Newly identified ischemic lesions confirmed by cranial computed tomography (CT) or magnetic resonance imaging (MRI) within 72 h post-surgery. Clinical neurologic outcomes (TND and PND) were assessed separately using standardized clinical evaluation (e.g., daily neurological examination by the intensive care team and neurologists.

#### 2.3.7 Postoperative respiratory failure

Defined by meeting ≥1 criterion within 24 h.

Arterial partial pressure of oxygen (PaO₂) < 60 mmHg while breathing room air.

Arterial partial pressure of carbon dioxide (PaCO₂) > 50 mmHg.

PaO₂/fraction of inspired oxygen (FiO₂) ratio <300 mmHg (7).

#### 2.3.8 Thromboembolic events

Diagnosed via combined duplex ultrasonography of extremities/neck and elevated D-dimer (>0.5 μg/mL) (8).

#### 2.3.9 Postoperative re-sternotomy

Due to excessive drainage and unstable circulation, re-sternotomy was required for exploration and hemostasis.

#### 2.3.10 Postoperative hoarseness

Attributed to recurrent laryngeal nerve injury during dissection of aortic arch branches.

### 2.4 Key covariates

#### 2.4.1 Deep hypothermic circulatory arrest (DHCA) duration

Circulatory arrest time (minutes) maintained at nasopharyngeal temperature ≤20.0 °C (9).

#### 2.4.2 Preoperative stroke history

Documented cerebrovascular event with supporting preprocedural CT/MRI evidence.

#### 2.4.3 Hypertension severity

Classified per WHO criteria:

Grade I: Systolic blood pressure (SBP) 140–159 mmHg and/or diastolic blood pressure (DBP) 90–99 mmHg.

Grade II: SBP 160–179 mmHg and/or DBP 100–109 mmHg.

Grade III: SBP ≥180 mmHg and/or DBP ≥110 mmHg (10).

#### 2.4.4 Involved sites of dissection

Aortic valve apparatus or coronary ostia.

Renal/mesenteric arteries.

Lower limb malperfusion (documented by absent dorsalis pedis pulse or ischemic signs).

#### 2.4.5 Preoperative cardiac tamponade

Hemodynamically significant pericardial effusion requiring emergent intervention.

#### 2.4.6 Surgical strategy

Isolated ascending aortic replacement.

Total arch replacement with frozen elephant trunk (TAR + FET) implantation.

### 2.5 Details of perfusion cannulation procedure

#### 2.5.1 Innominate artery antegrade perfusion (IA)

Innominate artery anastomosis antegrade perfusion operation method: Dissect the branches of the aortic arch, make a longitudinal incision of about 0.8–1 cm with a surgical scalpel 2 cm above the origin of the innominate artery, clamp both ends of the innominate artery incision with occlusion clamps, take a 1 cm diameter artificial straight vessel, and perform an end-to-side anastomosis to suture the artificial straight vessel onto the innominate artery. After suturing, connect the other end of the artificial straight vessel to an arterial cannula, and secure it with multiple loops of #10 silk suture. Insert a caval drainage tube into the right atrium to establish extracorporeal circulation. When deep hypothermic circulatory arrest is required for distal aortic manipulation, clamp the proximal end of the innominate artery anastomosis with an occlusion clamp to maintain unilateral cerebral perfusion. After completing the distal aortic manipulation, if total arch replacement is performed, prioritize the anastomosis of the left common carotid artery to restore bilateral cerebral perfusion as soon as possible. After the completion of extracorporeal circulation and neutralization with protamine, when removing the arterial cannula, it is only necessary to place three Hemolok clips at 0.5 cm above the anastomosis of the innominate artery and then cut the distal end.

#### 2.5.2 Combined perfusion of the femoral artery and innominate artery (IA + FA)

Incision of the femoral artery, isolation of the femoral artery, and after completion of femoral artery cannulation, connect the innominate artery to another arterial cannula using the aforementioned method to provide cerebral protection.

#### 2.5.3 Prerequisites and protocol for innominate artery cannulation

The decision to proceed with innominate artery (IA) antegrade cannulation was contingent upon a thorough preoperative and intraoperative assessment. Suitability for IA cannulation was primarily determined by preoperative computed tomography angiography (CTA), which was reviewed to ensure the following criteria were met: (1) the origin and proximal segment of the innominate artery were free of significant dissection flap involvement; (2) there was an absence of severe calcification or atherosclerotic plaque at the intended cannulation site (approximately 2 cm above the artery's origin); (3) the vessel diameter was deemed adequate for safe cannulation with an 8–10 Fr arterial cannula.

If preoperative CTA suggested potential dissection extension into the innominate artery, the surgical strategy was reconsidered. In cases where the dissection flap was limited and a satisfactory true lumen could be confidently identified intraoperatively, cannulation might still be attempted under direct vision, ensuring the cannula was placed securely within the true lumen. However, if significant involvement, complex tear, or poor true lumen identification was confirmed, the IA cannulation strategy was abandoned. In such scenarios, the protocol was to utilize the combined IA + FA approach, relying on the femoral artery as the primary arterial inflow for systemic perfusion while using the innominate artery connection primarily for unilateral cerebral protection during circulatory arres.

#### 2.5.4 Flow distribution and circuit setup

During cardiopulmonary bypass (CPB) establishment for the IA + FA strategy, a dual arterial line configuration was employed. The femoral artery cannula served as the primary inflow for systemic perfusion, connected to the main pump head of the CPB circuit. The innominate artery cannula was connected to a separate, independent auxiliary pump head, dedicated specifically to antegrade cerebral perfusion.

Target Flow Rates and Monitoring:

The target flow rates were as follows:

Systemic perfusion (via femoral artery): Aimed to maintain a systemic perfusion index of 2.2–2.4 L/min/m^2^ during moderate hypothermia (25–28 °C), with a target mean arterial pressure of 50–70 mmHg.

Cerebral perfusion (via innominate artery): Provided unilateral antegrade cerebral perfusion at a flow rate typically set between 8 and 12 mL/kg/min (approximately 400–800 mL/min). The adequacy of cerebral perfusion was assessed by monitoring right radial artery pressure and, when available, guided by intraoperative neuromonitoring such as near-infrared spectroscopy (NIRS).

Intended Mixing Zone:

The blood from the two antegrade perfusion sites (innominate and femoral arteries) was intended to mix within the distal aortic arch and descending aorta. This strategy aimed to provide perfusion to the lower body, including the visceral organs (e.g., renal, mesenteric arteries). Throughout the CPB run, the perfusionist closely monitored arterial pressures, venous oxygen saturation, and cerebral oximetry (if used), making fine adjustments to the flow rates of both pump heads to ensure adequate end-organ perfusion while avoiding excessive pressure that could cause false lumen expansion.

### 2.6 Statistical analysis

All data were analyzed using SPSS software (version 26.0).

#### 2.6.1 Categorical variables were analyzed using Pearson’s chi-square test (*χ*^2^) (for expected frequencies ≥5) or Fisher’s exact test (for sparse cells with expected frequencies <5)

#### 2.6.2 Continuous variables are presented as mean ± standard deviation (SD)

Group comparisons employed:

Independent samples t-test for normally distributed data.

Mann–Whitney U test (rank-sum test) for non-normally distributed data.

#### 2.6.3 A two-sided *p*-value <0.05 was considered statistically significant

#### 2.6.4 Given the sample size and the rates of observed events, a *post-hoc* power analysis was performed for the primary endpoint

#### 2.6.5 Multivariate logistic regression analysis for 30-day mortality

## 3 Results

### 3.1 Patient characteristics and preoperative data

A total of 94 patients who underwent surgical repair for ATAAD were included in the final analysis and allocated into two groups based on the perfusion strategy: the IA group (*n* = 56) and the IA + FA group (*n* = 38).

The baseline demographic, clinical, and operative characteristics of the two groups are summarized in Table 1. Preoperative comparisons revealed no statistically significant differences between the two groups. The cohorts were well-matched in terms of age (58.71 ± 14.54 vs. 55.58 ± 14.64 years, *p* = 0.31), BMI (24.56 ± 3.3 vs. 25.46 ± 3.17 kg/m^2^, *p* = 0.19), and sex distribution (male: 78.57% vs. 78.95%, *p* = 1.00). Furthermore, the prevalence of major comorbidities, including hypertension (grades I-III), smoking status, diabetes mellitus, and a history of preoperative cerebral infarction, was comparable between groups (all *p* > 0.05).

**Table 1:** Baseline and preoperative characteristics.

Characteristic	Overall (*n* = 94)	IA group (*n* = 56)	IA + FA group (*n* = 38)	*p*-value
Demographics				
Age, years	57.45 ± 14.58	58.71 ± 14.54	55.58 ± 14.64	0.310
Male sex	74 (78.7)	44 (78.6)	30 (78.9)	1.000
BMI, kg/m^2^	24.93 ± 3.26	24.56 ± 3.30	25.46 ± 3.17	0.190
Comorbidities				
Smoking history	12 (12.8)	8 (14.3)	4 (10.5)	0.830
Hypertension	68 (72.3)	40 (71.4)	28 (73.7)	0.200
Diabetes mellitus	4 (4.3)	2 (3.6)	2 (5.3)	1.000
Preoperative Status				
Cardiac tamponade	30 (31.9)	20 (35.7)	10 (26.3)	0.460
Cardiogenic shock	16 (17.0)	11 (19.6)	5 (13.2)	0.590
Preoperative coma	6 (6.4)	3 (5.4)	3 (7.9)	0.950
Dissection extent				
Aortic sinus involvement	44 (46.8)	26 (46.4)	18 (47.4)	1.000
Coronary artery involvement	6 (6.4)	5 (8.9)	1 (2.6)	0.240
Renal artery involvement	8 (8.5)	6 (10.7)	2 (5.3)	0.460

Data presented as mean ± standard deviation or *n* (%). BMI, body mass index.

*P*-value calculated using independent t-test for continuous variables and Chi-square or Fisher's exact test for categorical variables.

Critically, the groups were also balanced with respect to the severity and anatomical complexity of the aortic dissection. There were no significant differences in the incidence of preoperative coma (5.36% vs. 7.89%, *p* = 0.95), cardiac tamponade (35.71% vs. 26.32%, *p* = 0.46), or involvement of key aortic segments and branch vessels, including the aortic sinus (46.43% vs. 47.37%, *p* = 1.00), renal arteries (*p* = 0.46), and coronary arteries (*p* = 0.24). This equilibrium in preoperative risk factors confirms the validity of the subsequent comparative analysis.

### 3.2 Operative efficiency and perfusion data

Analysis of operative metrics revealed a significant difference in surgical efficiency between the two strategies (Table 2).

**Table 2:** Operative data and efficiency metrics.

Variable	IA group (*n* = 56)	IA + FA group (*n* = 38)	*p*-value
Surgical duration, min	370.05 ± 63.34	397.26 ± 62.11	0.040
Aortic cross-clamp time, min	109.96 ± 28.67	109.82 ± 30.41	0.980
Deep hypothermic circulatory arrest, min（DHCA）	22.64 ± 7.16	20.92 ± 8.16	0.280
Total cardiopulmonary bypass time, min	172.54 ± 43.49	169.39 ± 44.40	0.730

Data presented as mean ± standard deviation. *P*-value calculated using independent t-test.

Surgical Duration: The total operative time was significantly shorter in the IA group compared to the IA + FA group (370.05 ± 63.34 min vs. 397.26 ± 62.11 min, *p* = 0.04).

Aortic Cross-Clamp and Circulatory Arrest: In contrast, the durations of aortic cross-clamp (109.96 ± 28.67 vs. 109.82 ± 30.41 min, *p* = 0.98) and deep hypothermic circulatory arrest (22.64 ± 7.16 vs. 20.92 ± 8.16 min, *p* = 0.28) were nearly identical between the two groups.

### 3.3 Postoperative outcomes and complications

Postoperative outcomes are detailed in Table 3.

**Table 3:** Postoperative outcomes and recovery metrics.

Outcome	IA group (*n* = 56)	IA + FA group (*n* = 38)	*p*-value
Primary endpoint			
30-day mortality	6 (10.7)	8 (21.1)	0.280
Secondary endpoints			
Postoperative stroke	8 (14.3)	6 (15.8)	1.000
Transient neurologic deficit (TND)	6 (10.7)	5 (13.2)	0.750
Permanent neurologic deficit (PND)	2 (3.6)	1 (2.6)	1.000
Acute kidney injury	12 (21.4)	10 (26.3)	0.760
New hemodialysis requirement	7 (12.5)	9 (23.7)	0.260
Re-sternotomy for bleeding	2 (3.6)	4 (10.5)	0.360
Recovery Parameters			
Mechanical ventilation duration, h	91.30 ± 112.82	83.32 ± 103.83	0.730
Postoperative hospital stay, days	16.46 ± 12.05	15.00 ± 13.34	0.580
Laboratory Values (POD1)			
Creatinine, μmol/L	109.79 ± 74.11	129.74 ± 88.66	0.240
Lactate, mmol/L	4.31 ± 4.22	4.55 ± 4.75	0.800
Oxygenation index, mmHg	234.25 ± 136.17	255.21 ± 140.96	0.470

Data presented as mean ± standard deviation or *n* (%). POD1: postoperative day 1.

*P*-value calculated using independent t-test for continuous variables and Chi-square or Fisher's exact test for categorical variables.

Mortality: The 30-day all-cause mortality rate was 10.71% (6/56) in the IA group and 21.05% (8/38) in the IA + FA group, a difference that did not reach statistical significance (*p* = 0.28).

Organ-Specific Complications:

Neurological: The incidence of postoperative cerebral infarction was comparable between groups (14.29% vs. 15.79%, *p* = 1.00).The incidence of postoperative stroke (radiologically defined) and clinical neurologic deficits (TND and PND) were nearly identical between the two groups (*p* = 1.000 for stroke; *p* = 0.75 for TND; *p* = 1.00 for PND).

Renal: The rate of acute kidney injury (AKI) was not significantly different (21.43% vs. 26.32%, *p* = 0.76). However, a higher proportion of patients in the IA + FA group required postoperative hemodialysis (12.50% vs. 23.68%, *p* = 0.26).

Other Major Complications: No significant differences were found in the incidence of postoperative hoarseness (3.57% vs. 5.26%, *p* = 1.00), re-sternotomy for bleeding/tamponade (3.57% vs. 10.53%, *p* = 0.36), or arteriovenous thrombosis (7.14% vs. 5.26%, *p* = 1.00).

Recovery Parameters: Metrics of postoperative recovery, including duration of mechanical ventilation, ICU length of stay, and total postoperative hospital stay, showed no statistically significant differences between the two groups (all *p* > 0.05).

### 3.4 The distribution of surgical strategies between the two perfusion groups was analyzed

To assess the association between perfusion strategy and the extent of aortic replacement: In Table 4 the analysis revealed no statistically significant association between the perfusion method (IA vs. IA + FA) and the surgical strategy (isolated ascending replacement vs. total arch replacement with frozen elephant trunk), *χ*^2^(1, *N* = 94) = 3.734, *p* = 0.053.Regarding the surgical strategy, all patients underwent either isolated ascending aortic replacement or total arch replacement with frozen elephant trunk (TAR + FET) implantation. The distribution of these surgical strategies was comparable between the two perfusion groups (*p* = 0.053). In the IA group, 32 patients (57.1%) underwent isolated ascending replacement and 14 (25.0%) underwent TAR + FET. Corresponding numbers in the IA + FA group were 24 (63.2%) and 24 (63.2%), respectively.

**Table 4:** The distribution of surgical strategies between the two perfusion groups was analyzed.

Perfusion strategy	Isolated ascending replacement	Total arch + FET	*χ* ^2^	*P*-value
IA group	32	14	3.734	0.053
IA + FA group	24	24		
Total	56	38		

*P*-value calculated using independent chi-square test.

### 3.5 Multivariate logistic regression analysis for 30-day mortality

The model included the following covariates based on clinical relevance and literature: perfusion strategy (IA vs. IA + FA), age, sex, body mass index (BMI), history of preoperative coma, cardiac tamponade, surgical modus operandi (total arch replacement with frozen elephant trunk [TAR + FET] vs. isolated ascending replacement), total operative duration, total cardiopulmonary bypass time, aortic cross-clamp time, and deep hypothermic circulatory arrest (DHCA) time.

The detailed results of the multivariate analysis are shown in Table 5. After adjusting for all the aforementioned variables, the perfusion strategy (IA vs. IA + FA) was not an independent predictor of 30-day mortality [adjusted odds ratio [aOR] = 2.86, 95% confidence interval [CI]: 0.69–13.27, *p* = 0.156].

**Table 5:** Multivariate logistic regression analysis for 30-Day mortality.

VariableName	Estimate	Std_Error	Z_value	Pvalue	CI_2.5	CI_97.5	OR	Lower_OR	Upper_OR
(Intercept)	−8.4277	5.7486	−1.4660	0.1426	−20.9186	2.1362	0.0002	0.0000	8.4674
age	0.0270	0.0287	0.9409	0.3468	−0.0261	0.0898	1.0274	0.9742	1.0939
sex	−0.5485	1.0738	−0.5108	0.6095	−2.8763	1.4542	0.5778	0.0563	4.2811
BMI	0.1373	0.1505	0.9125	0.3615	−0.1497	0.4573	1.1472	0.8609	1.5797
Preoperative coma	1.0842	1.7565	0.6173	0.5371	−2.8445	4.3281	2.9572	0.0582	75.8022
Cardiac tamponade	1.1016	0.8088	1.3620	0.1732	−0.4694	2.7797	3.0089	0.6254	16.1147
Surgical modus operandi	1.8666	1.0925	1.7085	0.0875	−0.1732	4.1673	6.4666	0.8409	64.5414
Perfusion strategy	1.0504	0.7408	1.4179	0.1562	−0.3736	2.5857	2.8588	0.6883	13.2723
Total operative duration	−0.0045	0.0086	−0.5205	0.6027	−0.0218	0.0131	0.9955	0.9785	1.0132
Total cardiopulmonary bypass time	0.0144	0.0152	0.9470	0.3437	−0.0151	0.0459	1.0145	0.9850	1.0470
Aortic cross-clamp time	−0.0633	0.0289	−2.1893	0.0286	−0.1285	−0.0126	0.9386	0.8794	0.9875
DHCA	0.2214	0.1212	1.8267	0.0677	0.0112	0.4854	1.2478	1.0113	1.6248

Q-value calculated using Multivariate Logistic Regression Analysis.

OR, Odds Ratio; CI, Confidence Interval; CPB, Cardiopulmonary Bypass; DHCA, Deep Hypothermic Circulatory Arrest; TAR + FET, Total Arch Replacement with Frozen Elephant Trunk.

Among the other covariates included in the model, longer aortic cross-clamp time was significantly associated with a lower risk of mortality (aOR = 0.94 per minute increase, 95% CI: 0.88–0.99, *p* = 0.029). Conversely, longer DHCA time showed a trend towards an increased risk of mortality (aOR = 1.25 per minute increase, 95% CI: 1.01–1.62, *p* = 0.068). The extent of aortic replacement (TAR + FET vs. isolated ascending replacement) also approached but did not reach statistical significance (aOR = 6.47, 95% CI: 0.84–64.54, *p* = 0.088). No other variables, including age, sex, BMI, preoperative coma, cardiac tamponade, total operative duration, or total cardiopulmonary bypass time, demonstrated a statistically significant independent association with 30-day mortality in this model (all *p* > 0.05).

## 4 Discussion

This single-center retrospective study provides a comparative analysis of two predominant perfusion strategies—isolated innominate artery antegrade perfusion (IA) and combined innominate and femoral artery perfusion (IA + FA)—in the surgical repair of acute type A aortic dissection (ATAAD). The principal findings can be summarized as follows: (1) The IA strategy was associated with a statistically significant reduction in total operative time compared to the IA + FA approach. There were no significant differences in the primary endpoint of 30-day mortality or key secondary endpoints including postoperative stroke, acute kidney injury, and other major complications. Notably, the IA + FA group exhibited numerical trends towards higher rates of mortality, re-sternotomy, and requirement for hemodialysis, although these did not reach statistical significance in this cohort. These results collectively suggest that the IA perfusion strategy enhances surgical efficiency without compromising early clinical safety, thereby challenging the necessity of routine addition of femoral cannulation.

The most salient finding of our study is the significant improvement in surgical efficiency afforded by the IA strategy. The near 30-minute reduction in total operative time is clinically substantial, as prolonged duration in ATAAD surgery is independently associated with increased risks of bleeding, transfusion requirements, and surgical site infection. This finding is consistent with technical descriptions in the literature that have documented the additional time required for femoral vessel exposure and cannulation (11). Crucially, the fact that aortic cross-clamp and deep hypothermic circulatory arrest (DHCA) times were identical between groups indicates that this efficiency gain is not achieved by expediting the core aortic reconstruction. Instead, it is almost certainly attributable to the elimination of the time-consuming femoral artery dissection and cannulation procedure required in the IA + FA approach. This streamlined process reduces the complexity of establishing cardiopulmonary bypass, aligning with the surgical principle of minimizing unnecessary maneuvers in an already high-risk operation.

Regarding perioperative outcomes, our data demonstrates comparable safety profiles between the two strategies. The absence of a significant difference in the incidence of postoperative cerebral infarction (14.29% vs. 15.79%, *p* = 1.00) is particularly noteworthy. This finding robustly supports the physiological premise and previous guideline recommendations that antegrade cerebral perfusion via the innominate artery is sufficient for cerebral protection during DHCA (2, 12). It effectively mitigates the risk of retrograde embolization from atherosclerotic or dissected femoral and iliac vessels, a well-documented pitfall of femoral cannulation (12). Our stroke rates align with those reported in contemporary studies utilizing isolated antegrade perfusion strategies, and are notably lower than historical reports of retrograde perfusion techniques (13). The addition of femoral perfusion in the IA + FA group did not confer any detectable additional neurological benefit. Similarly, the rates of acute kidney injury, respiratory failure, and other organ dysfunctions were not significantly different, indicating that the IA strategy alone provides adequate systemic perfusion during the procedure.

However, the observed numerical trends towards worse outcomes in the IA + FA group warrant careful consideration. The higher 30-day mortality (21.05% vs. 10.71%), re-sternotomy rate (10.53% vs. 3.57%), and need for hemodialysis (23.68% vs. 12.50%), though not statistically significant in this sample, raise a crucial hypothesis. These trends are consistent with previous reports suggesting potential disadvantages of femoral cannulation (11). The technical complexity of dual cannulation may introduce unique risks, such as longer overall exposure to cardiopulmonary bypass, potential for atheroembolism from the femoral site, or iatrogenic lower limb vascular complications, which could counteract its theoretical benefits. This aligns with existing controversies in the literature regarding the potential for systemic embolization with combined techniques (4). A larger, multi-center study would be required to determine if these trends represent a true increased risk associated with the IA + FA approach.

The strengths of this study include a well-matched patient cohort, as evidenced by the homogeneity of all recorded baseline and preoperative risk factors, which strengthens the validity of our comparative outcomes. Furthermore, all surgeries were performed by a consistent surgical team following a standardized protocol, minimizing variations in technical expertise and operative philosophy.

This study found that the isolated innominate artery antegrade perfusion (IA) strategy significantly reduced the total operative time compared to the combined perfusion (IA + FA) strategy (370.05 ± 63.34 min vs. 397.26 ± 62.11 min, *p* = 0.04). We acknowledge that, under ideal conditions, dual-team simultaneous operation could partially overlap the preparation times for the femoral and innominate arteries. However, the time difference observed in this study may not merely be a result of sequential steps but rather a comprehensive reflection of the inherent complexity of the dual cannulation (IA + FA) strategy itself. This complexity could influence outcomes through multiple mechanisms beyond simply prolonging operative time. First, establishing and maintaining two independent arterial perfusion lines may prolong the patient's overall exposure time to cardiopulmonary bypass, which is associated with increased risks of inflammatory response and organ injury. Second, femoral artery cannulation itself introduces the risk of atheroembolism from the femoral site, particularly prominent in patients with extensive peripheral vascular disease, and may be associated with systemic embolic complications. Finally, the additional femoral artery manipulation increases the potential for iatrogenic lower limb vascular injury, bleeding, or local complications. Therefore, the clinical significance of the efficiency advantage offered by the IA strategy may lie not only in the absolute minutes saved but also in simplifying the perfusion pathway, thereby avoiding a series of potential risks associated with femoral artery cannulation. The trends toward worse outcomes in the IA + FA group in this study—including 30-day all-cause mortality (21.05% vs. 10.71%), re-sternotomy (10.53% vs. 3.57%), and new requirement for hemodialysis (23.68% vs. 12.50%)—although not statistically significant within this sample size, are consistent with this explanation and echo existing controversies in the literature regarding the potential risks of combined perfusion techniques. Future larger-scale studies are needed to distinguish the independent contributions of operative time versus the cannulation technique itself to patient outcomes.

Several limitations must be acknowledged. Firstly, the retrospective, non-randomized design inherits the potential for selection bias and unmeasured confounding factors, despite our efforts to control for known variables. Secondly, the sample size, while substantial for a single-center ATAAD study, may still be underpowered to detect small but clinically important differences in low-incidence complications (e.g., paraplegia, specific embolic events). The trends towards worse outcomes in the IA + FA group highlight the need for a larger, prospective analysis to definitively confirm or refute these signals. A *post-hoc* power analysis confirms that the study was underpowered to detect the observed difference in mortality. Finally, as a single-institution experience, the generalizability of our findings may be influenced by local surgical preferences and patient demographics. This study did not perform routine preoperative assessment of the integrity of the Circle of Willis, nor did it conduct subgroup analyses based on whether the left common carotid artery was involved or the presumed status of collateral circulation. Future studies incorporate detailed assessments of cerebrovascular anatomy (e.g., evaluation of the Circle of Willis via CTA) and cerebral perfusion (e.g., via near-infrared spectroscopy) to further clarify the safety boundaries of isolated innominate artery perfusion in different patient subgroups.

The fundamental goal of arterial cannulation in ATAAD surgery is to establish true-lumen CPB flow, regardless of the cannulation route; the choice of cerebral perfusion technique (e.g., selective antegrade cerebral perfusion via innominate artery, retrograde cerebral perfusion) should be considered as an independent component of the overall perfusion strategy, not necessarily tied to the arterial inflow site.

While no significant difference was found in aortic cross-clamp or total CPB time—consistent with the premise that these are determined by the aortic pathology and reconstruction technique—the shorter total operative time in the IA group is logically attributable to the streamlined setup. The IA + FA approach necessitates additional steps for femoral artery access and management of a dual-perfusion circuit, which cumulatively extend the pre- and post-CPB phases of the operation without altering the duration of the central aortic repair itself.

While radiological infarction was used as a sensitive and objective marker, it may not equate to clinical significance. We will emphasize that the consistent, non-significant trends observed in both radiological and more clinically relevant functional outcomes (TND/PND) strengthen the conclusion that the IA strategy did not confer a higher neurological risk. We will also recommend that future studies prioritize standardized clinical assessment scales (e.g., NIH Stroke Scale, modified Rankin Scale at discharge) as primary endpoints.

## 5 Conclusion

In conclusion, our analysis demonstrates that for surgical repair of ATAAD, an isolated innominate artery antegrade perfusion strategy significantly improves surgical efficiency without a statistically significant increase in 30-day mortality or major complications compared to a combined IA + FA approach. These findings suggest that IA may be considered as a streamlined alternative perfusion strategy, potentially reserving IA + FA for select cases. Definitive conclusions regarding comparative survival outcomes require validation through larger, prospective studies, ideally with a non-inferiority design. Emphasize that for most ATAAD patients, a single true-lumen antegrade perfusion strategy (e.g., IA) is efficient and safe, while cerebral perfusion can be planned independently (e.g., via IA for SCP). Combined femoral artery perfusion should be reserved for specific indications.

## Data Availability

The original contributions presented in the study are included in the article/Supplementary Material, further inquiries can be directed to the corresponding authors.
